# Activation of Protein Kinase C Delta following Cerebral Ischemia Leads to Release of Cytochrome *C* from the Mitochondria via Bad Pathway

**DOI:** 10.1371/journal.pone.0022057

**Published:** 2011-07-15

**Authors:** Kunjan R. Dave, Sanjoy K. Bhattacharya, Isabel Saul, R. Anthony DeFazio, Cameron Dezfulian, Hung Wen Lin, Ami P. Raval, Miguel A. Perez-Pinzon

**Affiliations:** 1 Cerebral Vascular Disease Research Center, Department of Neurology, Leonard M. Miller School of Medicine, University of Miami, Miami, Florida, United States of America; 2 Neuroscience Program, Leonard M. Miller School of Medicine, University of Miami, Miami, Florida, United States of America; 3 Bascom Palmer Eye Institute, Leonard M. Miller School of Medicine, University of Miami, Miami, Florida, United States of America; 4 Department of Medicine, Leonard M. Miller School of Medicine, University of Miami, Miami, Florida, United States of America; Julius-Maximilians-Universität Würzburg, Germany

## Abstract

**Background:**

The release of cytochrome *c* from the mitochondria following cerebral ischemia is a key event leading to cell death. The goal of the present study was to determine the mechanisms involved in post-ischemic activation of protein kinase c delta (δPKC) that lead to cytochrome *c* release.

**Methods/Findings:**

We used a rat model of cardiac arrest as an *in vivo* model, and an *in vitro* analog, oxygen glucose deprivation (OGD) in rat hippocampal synaptosomes. Cardiac arrest triggered translocation of δPKC to the mitochondrial fraction at 1 h reperfusion. In synaptosomes, the peptide inhibitor of δPKC blocked OGD-induced translocation to the mitochondria. We tested two potential pathways by which δPKC activation could lead to cytochrome *c* release: phosphorylation of phospholipid scramblase-3 (PLSCR3) and/or protein phosphatase 2A (PP2A). Cardiac arrest increased levels of phosphorlyated PLSCR3; however, inhibition of δPKC translocation failed to affect the OGD-induced increase in PLSCR3 in synaptosomal mitochondria suggesting the post-ischemic phosphorylation of PLSCR3 is not mediated by δPKC. Inhibition of either δPKC or PP2A decreased cytochrome *c* release from synaptosomal mitochondria. Cardiac arrest results in the dephosphorylation of Bad and Bax, both downstream targets of PP2A promoting apoptosis. Inhibition of δPKC or PP2A prevented OGD-induced Bad, but not Bax, dephosphorylation. To complement these studies, we used proteomics to identify novel mitochondrial substrates of δPKC.

**Conclusions:**

We conclude that δPKC initiates cytochrome *c* release via phosphorylation of PP2A and subsequent dephosphorylation of Bad and identified δPKC, PP2A and additional mitochondrial proteins as potential therapeutic targets for ischemic neuroprotection.

## Introduction

The release of mitochondrial cytochrome *c* into the cytosol following cerebral ischemia was first observed in the late 1990's [1], [2], and is a key event that initiates the apoptotic cell death pathway while indirectly participating in the necrotic pathway leading to neuronal death [3]. Besides activating the apoptotic cascade, release of mitochondrial cytochrome *c* can also contribute to mitochondrial dysfunction including lower activity of mitochondrial respiratory chain complex IV [4]. The factors that contribute to mitochondrial cytochrome *c* release following cerebral ischemia include activation of pro-apoptotic Bcl-2 family of proteins (e.g Bad Bax, and Bak), opening of the mitochondrial permeability transition pore, activation of heat shock proteins, and increases in calcium levels, among others (see recent reviews for details: [5]). However, the precise mechanism by which cytochrome *c* is released from the mitochondria following cerebral ischemia is not fully understood [6].

Protein kinase c delta (δPKC) can initiate pro-apoptotic pathways by direct effects on the mitochondria [7], [8]. For example, δPKC phosphorylates mitochondrial phospolipid scramblase 3 (PLSCR3), resulting in increased cardiolipin expression on the mitochondrial outer membrane which facilitates apoptosis in HeLa cells [9]. Increased cardiolipin presence on the mitochondrial outer membrane recruits t-Bid (truncated BH3 interacting domain death agonist) which in turn results in formation of Bax/Bak pores through which cytochrome *c* release may occur [9], [10], [11]. Another target of δPKC phosphorylation is protein phosphatase 2A (PP2A) which may contribute to apoptosis by dephosphorylating Bad resulting in heterodimer formation and inactivation of Bcl-2 and Bcl-xL [12]. The inactivation of Bcl-2 and Bcl-xL permit the release of Bax which, when dephosphorylated by PP2A, can form a mitochondrial pore with Bak permitting cytochrome *c* release [11], [13]. Thus δPKC activation may result in phosphorylation of targets both within and outside the mitochondria resulting in cytochrome *c* release and apoptosis.

By utilizing *in vivo* and *in vitro* models of cerebral ischemia, we and others have demonstrated that δPKC is activated (translocated from soluble to the particulate membrane fraction) following cerebral ischemia [14], [15], [16]. When activation of δPKC is attenuated with a δPKC-specific peptide inhibitor (δV1-1), the brain is protected from ischemic neuronal damage [14], [15]. δPKC activation has been implicated in ischemia/reperfusion-induced injuries such as oxidative stress, apoptosis, and inflammation [7], [8], [17]. In previous studies, we observed that release of cytochrome *c* and activation of δPKC following cerebral ischemia were closely correlated [14], [15]. However, whether ischemia-induced δPKC translocation participates in the release of cytochrome *c* from mitochondria has not been defined.

The goal of the present study was to first test the hypothesis that δPKC translocation/activation after cerebral ischemia could result in cytochrome *c* release. We next tested two mechanistic hypotheses whereby δPKC may mediate cytochrome c release by: 1) phosphorylation/activation of PLSCR3 leading to targeting of t-Bid and downstream cytochrome *c* release, and 2) activation of δPKC phosphorylates PP2A leading to de-phosphorylation of pro-apoptotic factors Bad and Bax. Finally, we used proteomics to identify additional mitochondrial targets of δPKC which may be phosphorylated upon its translocation.

## Results

### δPKC translocates to the mitochondria following *in vivo* and *in vitro* cerebral ischemia

**Figure 1:**
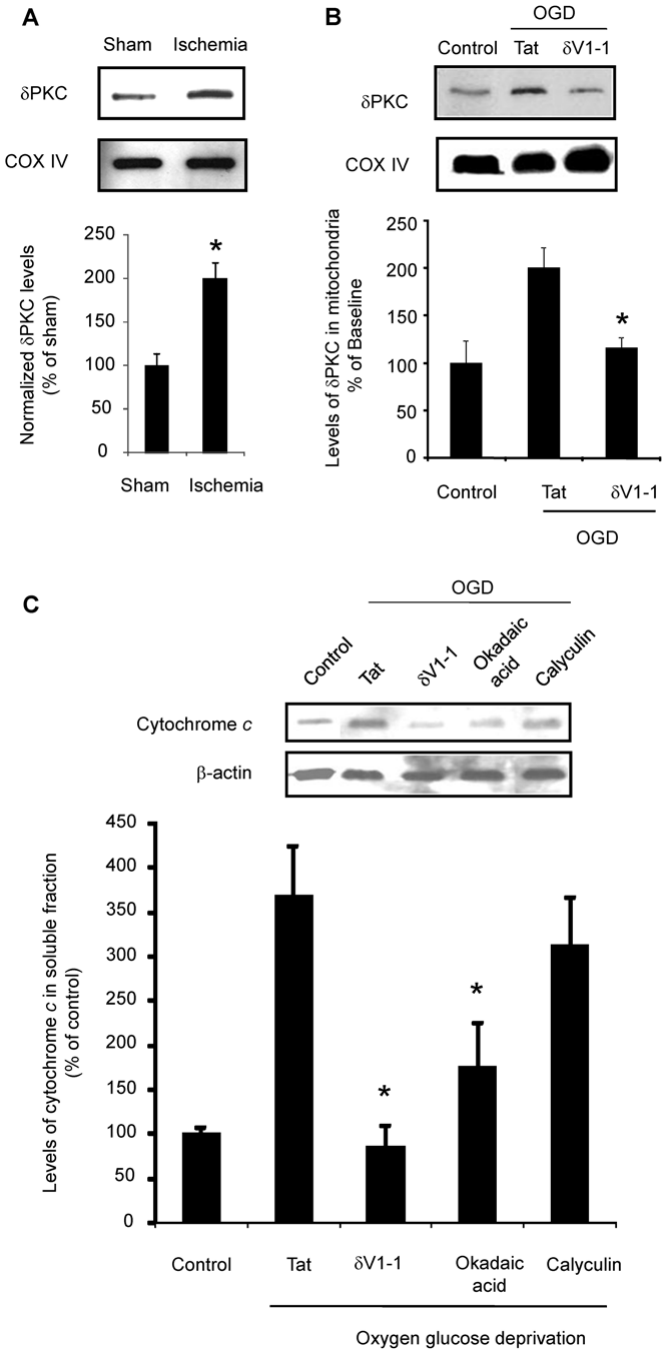
δPKC translocates to the mitochondria following CA and δPKC activation following OGD increases cytochrome *c* release. A) Immunoblot of δPKC in hippocampal mitochondria of a rat subjected to 8 min of CA and 1 h of reperfusion. Levels of δPKC normalized against COXIV for loading control are expressed as percentage from sham-operated (control) group. B) Immunoblots of δPKC in mitochondria isolated from hippocampal synaptosomes without ischemia or following 1 h oxygen glucose deprivation (OGD) in presence of tat (control) or δPKC inhibitor (δV1-1). Levels of δPKC normalized against COXIV for loading control are expressed as percentage of control δPKC protein expression. C) Immunoblot of cytochrome *c* in the soluble fraction of hippocampal synaptosomes subjected to oxygen glucose deprivation (OGD) in the presence of tat, δV1-1 or okadaic acid. Levels of cytochrome *c* normalized against β-actin for loading control are expressed as percentage of OGD in the presence of tat (control) group. *, *p*<0.05 v. sham or tat treated group.

We first tested the hypothesis that cerebral ischemia results in δPKC translocation from the cytosol to the mitochondria. Previously, we had observed in this model of cardiac arrest (CA) that cytochrome *c* is released at 1 h of reperfusion. Therefore, we examined δPKC translocation to hippocampal mitochondria at this time [15]. After 8 min of CA, hippocampal mitochondrial fraction of δPKC was enhanced by 99% (n = 4, *p*<0.05) as compared to sham-operated animals (Figure 1A). To test whether CA induced δPKC translocation is recapitulated in our *in vitro* system, we induced OGD in synaptosomes in the presence of tat carrier peptide (control vehicle for the δPKC inhibitor peptide) and compared it with control (no ischemia) synaptosomes (Figure 1B). OGD in synaptosomes doubled δPKC protein levels in the mitochondrial fraction (n = 5, *p*<0.05). This increase in δPKC translocation was reversed by 85% (n = 5, *p*<0.05) upon OGD induction in the presence of δV1-1. Thus, δPKC translocates to the mitochondria during the first hour of reperfusion following *in vivo* and *in vitro* hippocampal neuronal ischemia. The translocation was abolished *in vitro* using δV1-1.

### δPKC mediates the release of cytochrome *c* following OGD

Since we demonstrated δPKC translocation at 1 h of reperfusion when cytochrome *c* release plateaus, we next sought to determine if inhibition of translocation prevents cytochrome *c* release. Rat hippocampal synaptosomes underwent 60 min of OGD in the presence of δV1-1 or tat peptide with subsequent measurements of cytosolic cytochrome *c*. These levels were normalized to the cytosolic cytochrome *c* levels of control synaptosomes incubated in glucose containing solution with room air insufflation. OGD of the synaptosomes in the presence of tat peptide resulted in a 268% increase in cytochrome *c* release from the mitochondria (n = 5) as compared to control. Inhibition of δPKC translocation (via δV1-1) resulted in cytochrome *c* release similar to control and significantly less than untreated OGD synaptosomes (n = 5, p<0.05; Figure 1C). Thus, δPKC inhibition prevents cytochrome *c* release favoring neuronal survival.

### Phospholipid scramblase 3 (PLSCR3) phosphorylation following cerebral ischemia is not due to δPKC activation/translocation

**Figure 2:**
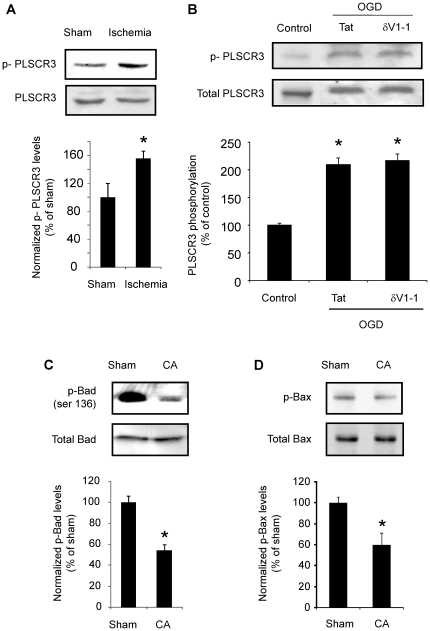
Phospholipid scramblase 3 (PLSCR3), Bad and Bax phosphorylation following cerebral ischemia. A) Immunoblot of p-PLSCR3 and PLSCR3 in hippocampal mitochondria of a rat subjected to 8 min of CA and 1 h of reperfusion. B) Immunoblot of p-PLSCR3 and PLSCR3 in synaptosomes subjected to oxygen glucose deprivation (OGD) in the presence of tat or δV1-1. Immunoblots (representative images are shown at the top of each bar) were subjected to densitometric analyses, and levels of p-PLSCR3 were normalized against PLSCR3 for loading control is expressed as percentage of OGD in the presence of tat (control) group. Immunoblot of (C) p-Bad and (D) p-Bax in hippocampal homogenate of a rat subjected to 8 min of CA and 1 h of reperfusion. Levels of p-Bad and p-Bax normalized against total-Bad and total-Bax, respectively are expressed as percentage of sham operated (control) group. *, *p*<0.01 v. sham. *, *p*<0.05 v. sham or tat treated group.

We hypothesized that post-ischemic δPKC phosphorylation of PLSCR3 could increase cardiolipin availability on the outer membrane of the mitochondria facilitating cytochrome *c* release through targeting of t-Bid and subsequent activation of Bax and Bak [10]. Indeed, CA led to a 56% (n = 4, p<0.05) increase in phospho-PLSCR3 (p-PLSCR3) in mitochondrial fraction as compared to sham-operated rats (Figure 2A) and an even larger increase in PLSCR3 phosphorylation (110% compared to control, p<0.05; Figure 2B) was noted *in vitro*. However, PLSCR3 phosphorylation did not change significantly when δPKC was inhibited during OGD (Figure 2B). Thus, δPKC activation/translocation was dissociated from phosphorylation of PLSCR3 and fails to mechanistically explain the increase in cytochrome *c* release following cerebral ischemia (Figure 1C).

### Protein Phosphatase 2A activation leads to release of cytochrome *c*

δPKC activates PP2A via phosphorylation [12] although it is unclear whether this is due to δPKC translocation to yet uncharacterized cytoskeletal receptors in close proximity to PP2A. PP2A has a number of known downstream targets, which we hypothesized could modulate cytochrome *c* release. To test this hypothesis, synaptosomes were subjected to 60 min of OGD in the presence of two protein phosphatase inhibitors in separate experiments: okadaic acid 0.1 nM) and calyculin (10 nM), which are potent inhibitors of both PP1 and PP2A [18]. The K_i_ of okadaic acid is 0.1 nM for PP2A vs. 10 nM for PP1, making it more specific for PP2A, whereas the K_i_ of calyculin is 10-fold higher for PP1 as compared to PP2A [18], [19]. Okadaic acid lowered OGD-induced cytochrome *c* release by 52% (n = 5, p<0.05) (Figure 1C) as compared to vehicle OGD group. In contrast, calyculin lowered OGD-induced cytochrome *c* release by an insignificant 15% (n = 5) (Figure 1C) as compared to vehicle OGD group. These results suggest that cerebral ischemia-induced PP2A activation triggers cytochrome *c* release most likely via dephosphorylation of downstream targets.

### PP2A activation leads to release of cytochrome *c* via Bad and Bax pathway

**Figure 3:**
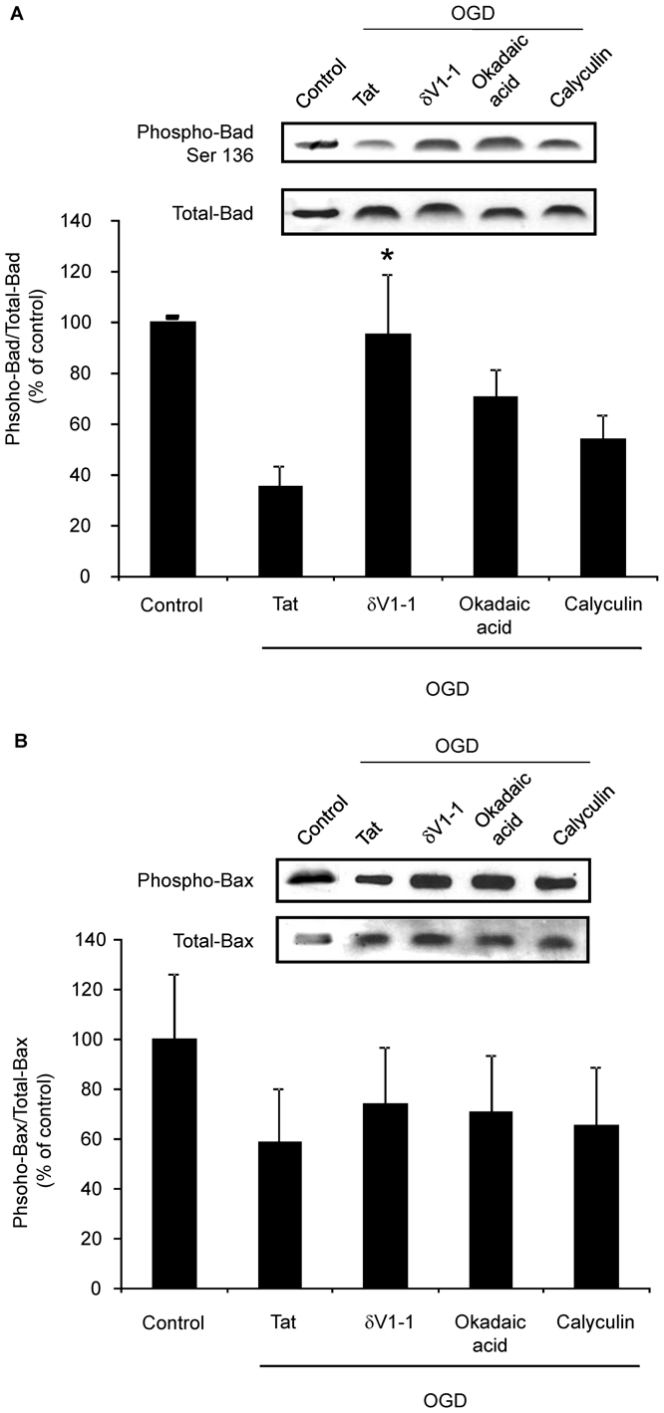
δPKC activation following OGD decreases Bad phosphorylation via PP2A activation. A) Immunoblot of p-Bad in hippocampal synaptosomes subjected to oxygen glucose deprivation (OGD) in the presence of tat, δV1-1 or okadaic acid. Immunoblots (representative images are shown at top of each bar) were subjected to densitometric analyses, and levels of p-Bad were normalized against total-Bad for loading control is expressed as percentage of OGD in the presence of tat (control) group. B) Immunoblot of p-Bax in hippocampal synaptosomes subjected to oxygen glucose deprivation (OGD) in the presence of tat, δV1-1 or okadaic acid. Immunoblots (representative images are shown at top of each bar) were subjected to densitometric analyses, and levels of p-Bax were normalized against total-Bax for loading control is expressed as percentage of OGD in the presence of tat (control) group. * *p*<0.05 v. tat treated group.

We next hypothesized that PP2A activation following δPKC-mediated phosphorylation could result in dephosphorylation of pro-apoptotic proteins Bad and Bax, both of which would be expected to result in increased cytochrome *c* release [20], [21]. The level of Bad and Bax phosphorylation was determined at 1 h of reperfusion in rat hippocampal homogenates subjected to CA (Figure 2C and D). We observed that the levels of phospho-Bad and phospho-Bax were decreased by 46% (n = 4, p<0.01) and 49% (n = 4, p<0.05), respectively as compared to sham-operated rats. To test whether the Bad and Bax de-phosphorylation observed *in vivo* resulted from δPKC induced phosphorylation/activation of PP2A, we tested the impact of synaptosomal OGD in the presence of either δV1-1 or the PP2A inhibitor okadaic acid (0.1 nM). Both inhibitors (δV1-1 and okadaic acid) decreased OGD-induced Bad de-phosphorylation (i.e. led to increased phosphorylation) by 170% (n = 5, p<0.05) and 101% (n = 5), respectively as compared to OGD in the presence of tat peptide group (Figure 3A). Calyculin had a less significant (54%) effect on Bad de-phosphorylation. However, the effect of all inhibitors on Bax phosphorylation was marginal and insignificant (Figure 3B). These results suggest that δPKC activation following cerebral ischemia phosphorylates PP2A resulting in subsequent Bad, but not Bax, de-phosphorylation ultimately resulting in increased release of cytochrome *c*.

### Additional substrates for δPKC within the mitochondria

**Figure 4:**
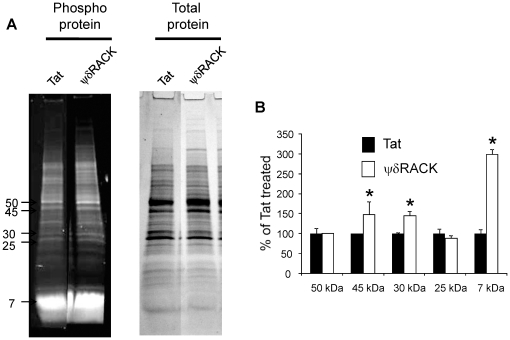
δPKC induced increased protein phosphorylation. Mitochondria were isolated from hippocampal synaptosomes treated with tat or ψδRACK. Mitochondrial proteins were separated on a 4–20% polyacrylamide gel. (A) Images of gels stained with ProQ diamond or Coomassie blue to detect phosphoproteins or total proteins, respectively. (B) Gel images were subjected to densitometric analysis for 5 bands (50 kDa, 45 kDa, 30 kDa, 25 kDa, and 7 kDa). Phosphoprotein signal was normalized against Coomassie blue stained gel signal. The results are expressed as percent of tat treated group. *, p<0.05 v. tat treated group.

Although it is established that δPKC initiates apoptosis via the mitochondrial pathway [15], [22], [23], until now only four mitochondrial substrates (d subunit of Fo-F1 ATPase, pyruvate dehydrogenase kinase -2, phospholipid scramblase 3 and acid sphingomyelinase) for δPKC have been identified. Since PLSCR3 did not appear to be an important mitochondrial target of translocated δPKC, we sought to identify additional potential mitochondrial substrates for δPKC. Additional phospho-proteins were identified in mitochondria isolated from synaptosomes treated with δPKC activator peptide (ψδRACK) or tat carrier peptide (control). δPKC can translocate to membrane/particulate fraction by ψδRACK induced δPKC activation [24]. Using the Pro-Q Diamond phospho-protein gel stain, we noticed distinct bands of molecular weight ∼45, 30 and 7 kDa which were enriched by 48% (p<0.05), 45% (p<0.05) and 199% (p<0.001) (n = 4 each) with ψδRACK treatment as compared to control (Figure 4). Protein identification was performed using MS/MS analysis which identified numerous mitochondrial proteins in the first two bands (∼45 and 30 kDa) which are listed in Table 1. Owing to technical challenges we were unable to identify proteins present in the third band (∼7 kDa). This list serves as a source for future investigations regarding mitochondrial targets of δPKC activated by cerebral ischemia.

**Table 1:** List of proteins identified in the proteomics study.

	Accession NCBI NR	Protein	MW kDa	Peptides identified	Score Xcalibur
**Band 45 kDa**	55741544	Ubiquinol cytochrome c reductase core protein 2	48	30	100.19
	58865384	NADH dehydrogenase (ubiquinone) Fe-S protein 2	53	14	70.26
	60678254	Creatine kinase, mitochondrial 1, ubiquitous	47	14	40.24
	158749584	Succinate-Coenzyme A ligase, beta subunit	50	7	40.21
	57657	Pyruvate dehydrogenase E1 alpha form 1 subunit	43	7	40.19
	54792127	Mitochondrial ATP synthase beta subunit	56	3	20.17
	149029485	ATP synthase, alpha subunit,	50	2	20.15
	18543177	Citrate synthase	52	3	10.15
**Band 30 kDa**	32189350	Solute carrier family 25, member 5	33	33	100.22
	6679299	Prohibitin	30	19	80.27
	47718004	Slc25a3 protein	40	11	60.20
	1580888	2116232A 2-oxoglutarate carrier protein	34	10	52.24
	157817227	NADH dehydrogenase (ubiquinone) Fe-S protein 3	30	10	40.21
	9507245	Tyrosine 3-monooxygenase	28	3	20.24
	157817027	Coiled-coil-helix-coiled-coil-helix domain containing 3	26	4	20.19
	59808764	Nipsnap1 protein	33	4	20.18
	51092268	NADH dehydrogenase (ubiquinone) flavoprotein 2	27	3	20.17
	89573817	Succinate dehydrogenase complex subunit B	27	3	20.13

## Discussion

**Figure 5:**
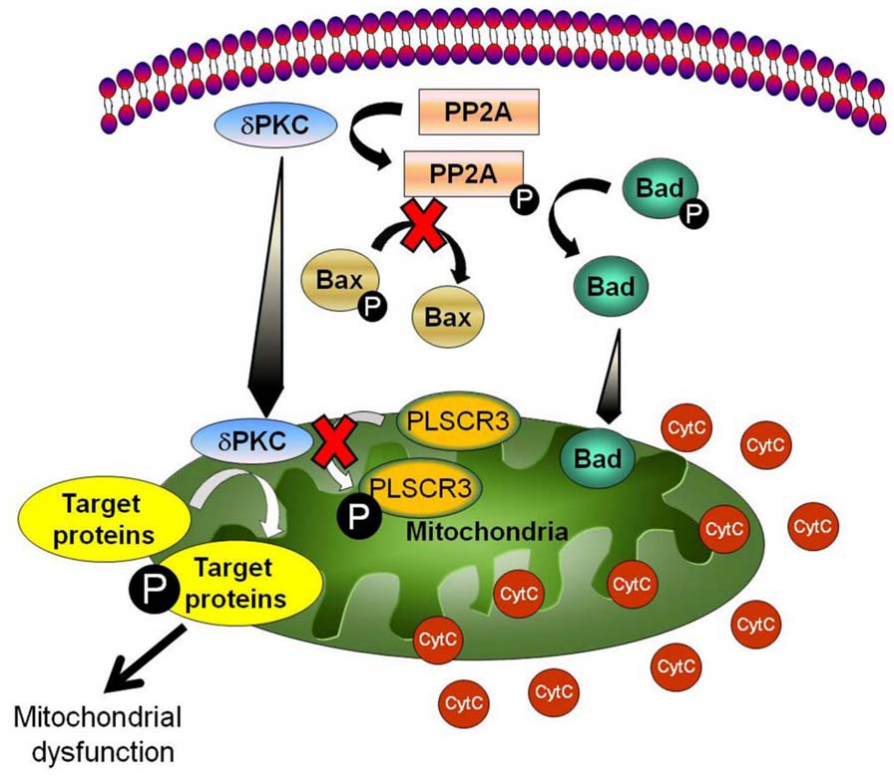
Schematic diagram of the mechanism by which cerebral ischemia-induced δPKC activation leads to cytochrome *c* release. Post-ischemic δPKC activation leads to activation of PP2A. Activated PP2A dephosphorylates Bad, but not Bax, which in turn release cytochrome *c* from mitochondria. In addition, δPKC can also contribute to mitochondrial dysfunction by phosphorylating other mitochondrial target proteins.

The precise signaling pathways that lead to cyotochrome *c* release from mitochondria after ischemia have not been fully elucidated. In the present study, we demonstrate that cerebral ischemia induces mitochondrial translocation of δPKC associated with cytochrome *c* release and that inhibition of δPKC translocation prevents this pro-apoptotic event. We next evaluated 4 distinct mechanistic hypotheses to explain this finding as summarized in Figure 5.

We had initially hypothesized that activated mitochondrial δPKC would phosphorylate PLSCR3 thereby increasing outer membrane cardiolipin which would recruit t-Bid and subsequently Bax and Bak, permitting pore formation and cytochrome *c* extrusion [10]. However, our results do not support this mechanism. Rather, we have identified a number of additional mitochondrial targets of δPKC translocation/activation which may explain the mechanism of cytochrome *c* release and apoptosis. Since δPKC is capable of targeting additional proteins outside the mitochondria, we chose to examine a pathway known to be regulated in neurons by δPKC and important in the control of apoptosis. Our results demonstrate that cerebral ischemia dephosphorylates Bad and Bax, events that promote apoptosis through formation of a mitochondrial pore to permit the release of cytochrome *c*. We demonstrate that the dephosphorylation of Bad but not Bax appears to be under the control of δPKC and PP2A activation.

In the present study, we used asphyxial CA in rats to model cerebral ischemia and demonstrated at each step that *in vitro* OGD using the synaptosome preparation recapitulates our *in vivo* results. Synaptosomes are considered to be the simplest possible anucleated, neuronally derived mammalian “mini-cell” (see review for details [25]). Synaptic mitochondria play a significant role in neurotransmitter release by regulation of cytosolic calcium [26] and thus would be expected to be highly susceptible to cerebral ischemia/reperfusion. In the present study, we observed that δPKC translocated to the synaptic mitochondria with *in vitro* ischemia and that this process could be efficiently inhibited with δV1-1. Since synaptic mitochondria are neuronally derived (excluding mitochondria from glia and neuronal body), we are limited in our ability to extrapolate our findings to the whole brain.

In earlier studies we reported that δPKC is activated (translocated from soluble to particulate fraction) following cerebral ischemia [14], [15]. In the present study, we demonstrate that mitochondria are one of the particulate fractions to which δPKC translocates following cerebral ischemia. This translocation may be initiated by the formation of reactive oxygen species, reactive nitrogen species and diacylglycerol formation [27]. Non-specific translocation of δPKC to mitochondria as a result of the isolation procedure may be ruled out due to the presence of lower levels of δPKC in shams *in vivo* and control synaptosomes *in vitro*. Oxidative stress-induced translocation of δPKC to mitochondria has been shown to be associated with loss of mitochondrial membrane potential and mitochondrial cytochrome *c* release [8], [28], [29], [30]. In the heart, ischemia/reperfusion results in diminished mitochondrial respiration which is restored by reperfusion in the presence of δPKC inhibitor. This contention is supported by our earlier study where we found that suppressed rate of respiration in presence of complex IV substrates at early reperfusion (30 min) following global cerebral ischemia was due to the release of mitochondrial cytochrome *c* [4]. We observed similar results using a rat model of asphyxial cardiac arrest, where we found that mitochondrial cytochrome *c* was released at 1 h of reperfusion following 8 min of cardiac arrest [15]. The present study thus provides potential targets for δPKC whose translocation to mitochondria early after ischemia is associated with mitochondrial dysfunction and mitochondrial cytochrome *c* release.

Since we observed that OGD-induced cytochrome *c* release was prevented by inhibition of δPKC, we explored possible pathways by which cerebral ischemia-induced δPKC activation led to the release of cytochrome *c*. First we determined whether δPKC exerted its effect by direct interaction with mitochondrial targets. Our first candidate was the phospholipid scramblase – 3 (PLSCR3), a known mitochondrial target of δPKC phosphorylation [31], [32]. PLSCR3, a member of phospholipid scramblase family of proteins, transports cardiolipin from the inner to the outer membrane of the mitochondria. Cytochrome *c* is bound to the outer surface of the inner mitochondrial membrane by associating with cardiolipin [33]. PLSCR3 had also been identified as a regulator of cardiolipin *de novo* biosynthesis [34]. Previous studies demonstrated that cardiolipin and cytochrome *c* interaction is a critical factor determining the amount of cytochrome *c* release during apoptotic stimuli [35], [36]. It is possible that following cerebral ischemia, PLSCR3, modified by δPKC, may result in impaired cardiolipin – cytochrome *c* interaction leading to cytochrome *c* release.

We observed that PLSCR3 phosphorylation was indeed increased following *in vivo* cerebral ischemia. However, in our synaptosomal model, we were unable to block OGD-induced increase in PLSCR3 phosphorylation by δV1-1 (δPKC-specific inhibitor peptide) at the same dose that inhibited cytochrome *c* release. Although previous studies concluded that PLSCR3 can be phosphorylated by δPKC; however, these studies used either general PKC activators (phorbol ester) or CMV promoter-driven δPKC overexpression (supra-physiological levels of δPKC) [31], [32]. No information is available on the effect of δPKC on PLSCR3 phosphorylation under physiological levels of δPKC or specific activation of δPKC without affecting other PKC isoforms. It is plausible that δPKC phosphorylates PLSCR3 in other cell types or neuronal compartments outside the synaptosome, as suggested by these studies, or that other PKC isoforms participate in PLSCR3 phosphorylation with ischemia. Our results suggest that PLSCR3 phosphorylation does occur in hippocampus *in vivo* but that δPKC inhibition does not block this process based on our *in vitro* data. Limitations exist when extrapolating an *in vitro* model results to *in vivo* findings, even when the *in vitro* model correlates well with the *in vivo* findings as we have already demonstrated.

Because δPKC-induced cytochrome *c* release was independent of mitochondrial PLSCR3 phosphorylation, we next examined other mitochondrial targets for δPKC phosphorylation. We demonstrated that the phospho-protein signal of three protein bands were increased in mitochondria isolated from synaptosomes treated with ψδRACK. Proteomics analyses of these bands revealed the presence of the following phosphoproteins: ubiquinol cytochrome *c* reductase core protein 2, Fe-S protein 2, 3 and flavoprotein 2 of NADH dehydrogenase, creatine kinase, beta subunit of succinate-coenzyme A ligase, E1 alpha form 1 subunit of pyruvate dehydrogenase (PDH), alpha and beta subunits of ATP synthase, citrate synthase, and prohibitin [37], [38].

An earlier study using *in vivo* models of cardiac ischemia/reperfusion demonstrated that δPKC activation (translocation to mitochondria) was responsible for decreased pyruvate dehydrogenase (PDH) activity following ischemia/reperfusion [39]. Their results support the hypothesis that activated δPKC interacts with and phosphorylates pyruvate dehydrogenase kinase (PDK)-2, which in turn phosphorylates the alphaE1 subunit of PDH, resulting in lower PDH activity. Based on our results and the earlier study, we hypothesize that the E1 alpha subunit of PDH is phosphorylated after ischemia/reperfusion-induced δPKC activation resulting in lower PDH activity in the brain [40], [41], [42]. Earlier studies demonstrated that ischemia/reperfusion resulted in lower ATP synthase and citrate synthase activities in the brain [43], [44], [45]. It is possible that ischemia/reperfusion-induced phosphorylation of ATP synthase (alpha and/or beta subunit) and citrate synthase via δPKC activation may be responsible for lower activities of these two enzymes. However, further investigation is required to support this hypothesis.

Our results suggest that prohibitin could be one of the mitochondrial substrates for δPKC [46]. Prohibitins are present in inner membrane of the mitochondria and forms multimeric ring complexes. Prohibits regulates mitochondrial fusion by regulating processing of the dynamin-like GTPase OPA1 [46], [47]. It is possible that δPKC-induced prohibitin phosphorylation may be responsible for cerebral ischemia-induced mitochondrial fission and suppressed mitochondrial respiration.

Mitochondrial cytochrome *c* release and apoptosis are also modulated by proteins located outside the mitochondrion. A prime example are the bcl-2 family proteins such as Bad and Bax whose activation and mitochondrial translocation are shown to induce cytochrome *c* release following apoptotic stimuli after cerebral ischemia [48], [49], [50], [51], [52], [53]. One potential pathway by which these molecules are activated is by de-phosphorylation via phosphatase [20], [21] such as PP2A. Zhang and colleagues demonstrated that PP2A activity is enhanced up to 3.4 fold following cerebral ischemia [54]. PP2A can also be activated by δPKC-induced phosphorylation [12]. PP2A is present in cytosol, membrane, cytoskeleton, and nuclear compartments, while δPKC is present in cytosol and translocates to particulate/membrane/cytoskeleton fraction following activation [55], [56], [57]. In which sub-cellular compartments δPKC interacts with PP2A is not known. In the present study, we were able to inhibit cerebral ischemia-induced Bad dephosphorylation as well as cytochrome *c* release by inhibiting δPKC or PP2A but not PP1 during ischemia. The slight inhibition observed with calyculin may be the result of PP2A effects, although 10-fold less than PP1. Our findings, therefore, support our proposed mechanism that δPKC activation via phosphorylation of PP2A results in cytochrome *c* release through the dephosphorylation of Bad.

In conclusion, our study demonstrated that δPKC translocated to mitochondria following cerebral ischemia/OGD. Post-ischemic activation of δPKC was not responsible for increased PLSCR3 phosphorylation but may target other mitochondrial proteins resulting in mitochondrial dysfunction and/or cytochrome *c* release. δPKC activation following cerebral ischemia led to the release of mitochondrial cytochrome *c* via the PP2A – Bad pathway.

## Materials and Methods

### Animals and Induction of cardiac arrest

All animal procedures were carried out in accordance with the Guide for the Care and Use of Laboratory Animals published by the National Institutes of Health and approved by the Animal Care and Use Committee of the University of Miami (protocol # 09-050). For experiments with hippocampus from naïve animals, male Sprague Dawley rats weighing 250–300 g were used. They were sacrificed under isoflurane anesthesia and the hippocampi were dissected out for further analyses. Asphyxial cardiac arrest was induced as described earlier [58], [59]. Head and body temperature, and blood gases were maintained in the normal range throughout the experiment. Sham animals were exposed to isoflurane identical to the experimental groups. Rats were sacrificed at 1 h post-ROSC and the hippocampus was dissected out and used for further analysis.

### Preparation of homogenate and isolation of mitochondria from rat hippocampus

Hippocampal mitochondria were isolated as described earlier with minor modifications [4]. Total mitochondria were isolated from the last pellet using nitrogen compression [60]. All mitochondrial isolation procedures were carried out at 4°C. Hippocampal synaptosomes were isolated as described earlier [60], [61]. Percoll density gradient was used to separate synaptosomes. Oxygen glucose deprivation (an *in vitro* model of ischemia) was induced by incubating synaptosomes in glucose deprived medium as described earlier [60], [62]. During standardization, we induced OGD of 30, 60 and 90 min and measured cytochrome *c*. We used 60 min of OGD for all experiments since cytochrome *c* release reached plateau at 60 min (80%, 114% and 110% at 30, 60 and 90 min of OGD, respectively). At the end of 60 min of OGD, mitochondria were isolated from synaptosomes using a nitrogen bomb [63]. Control synaptosomes were incubated in glucose medium containing bubbled with air at 30°C.

### *Ex vivo* δPKC activation and inhibition

Inhibition of δPKC was induced by pre-incubating synaptosomes with either tat carrier peptide or δV1-1 (δPKC inhibitor) (1 µM final concentration) for 15 min at room temperature (KAI Pharmaceuticals Inc., South San Francisco, CA, USA) [64], [65]. Tat carrier peptide or δV1-1 was also present during OGD. Activation of δPKC was induced by pre-incubating synaptosomes with either tat carrier peptide or ψδRACK (δPKC activator) (1 µM final concentration) for 15 min at room temperature.

### Immunoprecipitation and Western blotting

Immunoprecipitation was carried out using rabbit polyclonal anti-PLS3 (Imgenex, San Diego, CA, USA) or rabbit polyclonal anti- Bax (Santa Cruz biotechnology, Santa Cruz, CA, USA) and protein A sepharose beads (Sigma, St. Louis, MO, USA) as per manufacturer's instructions. The resulting immunoprecipitate was used for immunoblotting (see below) with subsequent probing using mouse monoclonal anti-phosphothreonine (Cell Signaling Technology, Danvers, MA, USA) with normalization to total protein levels using the primary antibody. Antibodies used were rabbit polyclonal anti-δPKC (Calbiochem, Gibbstown, NJ, USA), mouse monoclonal anti-cytochrome *c* (BD Pharmingen, San Jose, CA, USA), mouse monoclonal anti-cytochrome *c* oxidase subunit IV (Invitrogen, Carlsbad, CA, USA), or mouse monoclonal anti-β-actin (Sigma, St. Louis, MO, USA) antibodies and species specific secondary antibodies. The digitized immunoblots were subjected to densitometric analyses [60].

### Phosphoprotein staining, sample extraction from gel, and mass spectrometry

Proteins were separated from mitochondrial samples on a 4–20% acrylamide gel (Invitrogen Corporation, Carlsbad, CA). Phosphoproteins in the gradient gel were identified using Pro-Q Diamond Phosphoprotein Gel Stain (Molecular Probes Inc., Carlsbad, CA) as per manufacturer's instructions and subsequently stained with Coomassie blue (Pierce Biotechnology, Rockford, IL) to confirm equal protein loading. For protein identification, gel slices were excised and digested *in situ* with sequencing-grade trypsin (Promega Biosciences, Inc., Madison, WI). Samples were then processed for protein identification as described earlier [66].

### Statistical analysis

The results are expressed as mean ± SEM. Statistical significance was determined with Student's t-test when there were two experimental groups. For more than two groups, statistical evaluation of the data was performed using ANOVA test, followed by Dunnett's post hoc test with *p*<0.05 considered significant.
